# COVID-19 and Cardiovascular Complications: A Follow-Up Study from Tertiary Center

**DOI:** 10.3390/v17101293

**Published:** 2025-09-24

**Authors:** Danijela Lepojević-Stefanović, Stefan Živković, Dragana Marković, Gorica Marić, Nataša Marković-Nikolić

**Affiliations:** 1 Cardiology Department, Clinic for Internal Diseases, University Clinical Hospital Center “Zvezdara”, Dimitrija Tucovića 161, 11000 Belgrade, Serbia; 2 Institute for Cardiovascular Diseases “Dedinje”, Heroja Milana Tepića 1, 11040 Belgrade, Serbia; 3 Institute of Epidemiology, Faculty of Medicine, University of Belgrade, 11000 Belgrade, Serbia; 4 Internal Medicine Department, Faculty of Medicine, University of Belgrade, Dr Subotić 8, 11000 Belgrade, Serbia

**Keywords:** cardiovascular disease, complications, mortality, cohort study, survival

## Abstract

(1) Background: In addition to its fatal outcomes, COVID-19 is associated with a spectrum of non-fatal complications that significantly influence clinical trajectories and quality of life. Cardiovascular complications, in particular, are of major clinical relevance and are recognized as key contributors to both short- and long-term morbidity and mortality. The aim of the present study was to evaluate the short-term and long-term effects of COVID-19 infection on patients with underlying cardiovascular diseases. (2) Methods: The prospective cohort study included a total of 99 consecutive subjects hospitalized due to moderate and severe forms of COVID-19 pneumonia in “Zvezdara”—University Medical Center in the period of 18 March–18 April 2021. (3) Results: During hospitalization, 47% of patients had some new cardiovascular events. A total of 10 patients died during hospital stay. The highest chance for the lethal outcome was seen in those with previously diagnosed coronary heart disease (B = 3.356, OR = 28.667 (95% CI 2.69–305.14), *p* = 0.005), heart failure (B = 3.056, OR = 21.250 (95% CI 3.36–134.56), *p* = 0.001) and increased potassium values (B = 2.639, OR = 14.000 (95% CI 2.65–73.88), *p* = 0.002). (4) Conclusions: Care strategies for patients who survived the acute episode of COVID-19 should include attention to cardiovascular disease. Our findings emphasize the need for continued optimization of strategies for primary prevention of SARS-CoV-2 infections as the best way to prevent long COVID and serious cardiovascular complications.

## 1. Introduction

The COVID-19 pandemic has had a profound impact on global health, characterized by significant variation in infection rates, mortality, and hospitalization across different regions and time periods. First identified in Wuhan, China, in December 2019, the virus rapidly disseminated worldwide, resulting in substantial morbidity and mortality.

According to the Global Burden of Disease Study, approximately 15.9 million excess deaths occurred globally in 2020 and 2021, reflecting both the direct and indirect consequences of the pandemic [1]. Similarly, the World Health Organization estimated approximately 14.83 million excess deaths, underscoring the considerable underreporting of COVID-19-related mortality in official statistics [2]. An alternative analysis suggested an even higher figure—around 18.2 million excess deaths globally during the same period [3]. In the United States, excess mortality was estimated to exceed 1.17 million deaths between March 2020 and February 2022, followed by a marked decline in 2023 [4,5]. These data highlight the extensive and heterogeneous impact of the pandemic on global mortality, shaped by regional inequities and evolving epidemiologic trends.

In addition to its fatal outcomes, COVID-19 is associated with a spectrum of non-fatal complications that significantly influence clinical trajectories and quality of life. In-hospital complications are common and span multiple organ systems, including respiratory, cardiovascular, neurological, renal, and gastrointestinal manifestations. A large cohort study conducted in the United Kingdom found that 49.7% of hospitalized patients experienced at least one complication, with cardiovascular complications occurring in 12.3% of cases [6].

Cardiovascular complications, in particular, are of major clinical relevance and are recognized as key contributors to both short- and long-term morbidity and mortality. The underlying pathophysiological mechanisms involve a complex interplay of systemic inflammation, thromboinflammatory responses, direct myocardial injury via angiotensin-converting enzyme 2 (ACE2)-mediated viral entry, endothelial dysfunction, and exacerbation of pre-existing atherosclerotic or structural cardiac conditions [7,8].

Acute cardiovascular manifestations associated with COVID-19 include myocarditis, acute coronary syndrome, new-onset or decompensated heart failure, cardiogenic shock, arrhythmias (particularly atrial fibrillation and ventricular tachyarrhythmias), and thromboembolic or cerebrovascular events such as cerebrovascular insult [8,9]. A large study conducted in the United States of America reported that approximately 11.4% of hospitalized COVID-19 patients experienced an acute cardiac event, with notably higher rates observed among individuals with pre-existing cardiovascular disease [10].

Beyond the acute phase, the long-term cardiovascular sequelae of COVID-19 remain a significant clinical concern. These include persistent arrhythmias, recurrent cerebrovascular events, pericarditis, myocarditis, ischemic and non-ischemic cardiomyopathies, chronic heart failure, and venous or arterial thromboembolic disease. Importantly, such complications have been reported even in individuals who were not hospitalized during the acute infection, suggesting that SARS-CoV-2 may cause subclinical myocardial or endothelial injury with prolonged consequences [11,12].

Population-based cohort studies have demonstrated a statistically significant increase in the risk of cardiovascular events among COVID-19 survivors, including myocardial infarction, ischemic cardiomyopathy, heart failure, arrhythmias, thromboembolism, and cerebrovascular diseases within 12 months post-infection [11,13]. Cardiovascular complications have also emerged as key determinants of in-hospital mortality among patients with COVID-19 pneumonia, emphasizing their prognostic significance [14].

The long-term impact on health-related quality of life is also substantial. Many patients report persistent post-acute symptoms such as exertional dyspnea, fatigue, chest pain, and palpitations, which may reflect ongoing cardiac inflammation, autonomic dysfunction, or reduced cardiac reserve [15]. These sequelae contribute to impaired physical functioning, increased healthcare utilization, and prolonged rehabilitation. The burden is particularly pronounced in individuals with pre-existing cardiovascular disease, but is not confined to this population.

Collectively, the available evidence supports the need for continued cardiovascular surveillance among COVID-19 survivors, particularly in those with persistent symptoms or high baseline cardiovascular risk. A multidisciplinary approach and structured post-COVID-19 care pathways are warranted to mitigate long-term adverse outcomes and optimize patient prognosis. Given all previously mentioned, the aim of the present study was to evaluate the short-term and long-term effects of COVID-19 infection on patients with underlying cardiovascular diseases who were hospitalized at the Cardiology Department and then evaluated again after a 3-year follow-up period.

## 2. Materials and Methods

### 2.1. Settings

The prospective cohort study was performed among all patients hospitalized due to COVID-19, who were admitted at the Cardiology Department at the “Zvezdara”—University Medical Center during the period 18 March–18 April 2021. Three years after hospitalization, all patients included in the study were evaluated again and asked about new cardiovascular events during follow-up. Prior to hospitalization, a COVID-19 diagnosis was confirmed in all patients by a positive polymerase chain reaction (PCR) test result in a reference laboratory in Belgrade, the Capital of Serbia. All hospitalized patients were treated according to the protocol for the treatment of patients with COVID-19 pneumonia version 11, approved by the Ministry of Health of the Republic of Serbia. A study was approved by the Ethics Board of “Zvezdara”—University Medical Center (Approval number: 07/08/2025).

### 2.2. Selection of Participants

All COVID-19 patients hospitalized at the Cardiology Department during the study period were included in the study. No specific inclusion or exclusion criteria were applied.

### 2.3. Data Collection

Sources of data for this research were the electronic medical database Heliant and the medical histories of patients. Patients for whom data were not available were contacted via telephone to come to the hospital. Information extracted included epidemiological, clinical, echocardiographic, and radiological characteristics, as well as the type of treatment administered and the clinical outcome of the patient. Data on death outcome were extracted from medical records for patients who died during the hospital stay, or collected by contacting their family via phone. The data also comprised information on demographic characteristics, previous illnesses/injuries, comorbidities, symptoms and signs of the disease, laboratory findings, lung computed tomography (CT), and administered treatment (antiviral, corticosteroid, and respiratory support).

### 2.4. Procedures and Criteria for Newly Diagnosed CVD Events

Heart ultrasound (Canon Toshiba Aplio 500 Platinum, Canon Medical Systems Corporation, Tochigi, Japan) was performed in patients with newly diagnosed acute cardiovascular complications—acute coronary syndrome, pulmonary thromboembolism, arrhythmia, or heart failure (66 patients, 66.7%). Acute myocardial infarction was defined as typical symptoms associated with new ischemic electrocardiogram (ECG) changes, increased levels of troponin with or without new wall motion abnormalities on echocardiography. A new-onset arrhythmia was defined as newly verified rhythm irregularities recorded on the ECG or during the ECG Holter monitoring. New-onset heart failure implied the appearance of symptoms and/or signs of heart failure that the patient has not had before: the appearance of lung congestion, distended neck veins, swelling of the legs, enlarged liver, structural or functional impairments on heart ultrasound, and an increase in brain natriuretic peptides (BNP-s) levels (for those for whom it was determined). New pulmonary embolism was defined by the sudden appearance of a feeling of shortness of breath or chest pain caused by an increase in D-dimer values above the reference values and direct visualization of the occlusion of the pulmonary artery and/or its branches on the CT (CANON Aquilion Prime SP, Canon Medical Systems Corporation, Tochigi, Japan) pulmonary angiography. Pulmonary hypertension was defined as an RVSP value higher than 35 mm Hg.

### 2.5. Outcomes

The primary endpoint in the study was a death outcome during hospitalization or during a follow-up period. During follow-up, symptoms and signs of long COVID, newly developed cardiovascular events, COPD, T2D, malignancies, hospitalizations, and mortality were evaluated.

### 2.6. Statistical Analysis

Numerical data were presented with arithmetic mean and standard deviation or median and range (min–max), depending on the data distribution. Testing the normality of the distribution was performed by mathematical (coefficient of variation, skewness, and kurtosis, and tests—Kolmogorov–Smirnov and Shapiro–Wilk) and graphical methods. Categorical variables were shown as absolute and relative numbers in the form *n* (%). The Chi-square test was used to compare nominal variables between two independent samples. To examine the influence of laboratory findings, ECG, and echocardiographic parameters, as well as existing cardiovascular events on the death outcome of the subjects, logistic regression analysis was used, and presented as the regression coefficient B, the Odds ratio (OR), ninety-five percent confidence interval for the OR (95% CI OR), and *p*-value. All statistical analytical methods were considered significant at a significance level of α = 0.05. The complete analysis was performed in the software package IBM SPSS Statistics for Windows, Version 21.0, Armonk, NY, USA, IBM Corp.

## 3. Results

### 3.1. Study Sample

This study included a total of 99 patients (62.2% of males and 37.8% of females, male-to-female ratio of 2:1). The average age of the participants was 68.6 ± 11.8 years. The prevalence of cardiovascular risk factors at hospital admission in all subjects included in the study is shown in Table 1. The most common were hypertension (present in 84% of cases), coronary artery disease (CAD) (34%), and type 2 diabetes (25.5%), while the rarest was deep vein thrombosis (2%).

Average values of systolic and diastolic blood pressure were 129.3 ± 17.8 mmHg and 78.9 ± 9.4 mmHg, respectively, and the mean heart rate was 77.7 ± 14.9. As can we observe in Table 2, the analyzed heart ultrasound parameters on 66 patients showed left and right heart dimensions within normal range, the majority of patients (*n* = 42) had preserved ejection fraction, and their right ventricular systolic pressure was within normal range.

Among the participants, 30.3% had unilateral pneumonia, while bilateral pneumonia was present in 69.7% of them. Basal and diffuse pneumonia were represented in the ratio 2:1, in favor of basal. Oxygen therapy was required in 96% of patients (in 45% high-flow oxygen therapy), and mechanical ventilation was performed in 14% of the study sample. Lung CT was performed in patients with clinical, respiratory, and laboratory indicators of disease progression—47% of the sample. The average value of the CT score as an indicator of the severity of pneumonia in these patients was 20.36 ± 2.44.

The medication treatment included antibiotics—either one or a combination—for 74% of patients, and antiviral therapy (Favipiravir or Remdesivir) for 30% of patients. Immunomodulatory therapy (inhibitor IL-6 or Actemra) was given to 31% of patients, while corticosteroid therapy was given in 89% of patients during hospitalization and 38% during the first two or three weeks after discharge. As we can see in Table 1, a very large number of patients had hypertension on admission, and even 52% had an ACE inhibitor in their regular therapy. Beta blockers with 64% were the most frequently administered cardiological drugs during the hospital stay. Patients with symptoms of heart failure, depending on the severity of symptoms and cause, received loop diuretics, MRA (mineralocorticoid receptor antagonists), antiarrhythmics, nitrates, antitrombotics, and different supportive therapies. SGLT inhibitors were administered in only two patients.

### 3.2. Cardiovascular Events

During hospitalization, 47% of patients experienced new cardiovascular events (Table 3). The most common was pulmonary embolism, present in 15% of patients, followed by atrial fibrillation, heart failure, and pericardial effusion.

A total of 10 patients died during their hospital stay. Table 4 contains the results of logistic regression analysis with significant predictors of intrahospital mortality. The highest chance for the lethal outcome was seen in those with previously diagnosed CAD (B = 3.356, OR = 28.667 (95% CI 2.69–305.14), *p* = 0.005), heart failure (B = 3.056, OR = 21.250 (95% CI 3.36–134.56), *p* = 0.001), and increased potassium values (B = 2.639, OR = 14.000 (95% CI 2.65–73.88), *p* = 0.002).

### 3.3. Follow-Up Period

When it comes to the prevalence of late COVID-19 symptoms, during the second measurement in 2024, extreme malaise and sleepiness were present in 21 (21.2) patients, with 11 (11.1%) of them reporting still having shortness of breath, and 9 (9.1%) had palpitations (Table 5).

**Figure 1:**
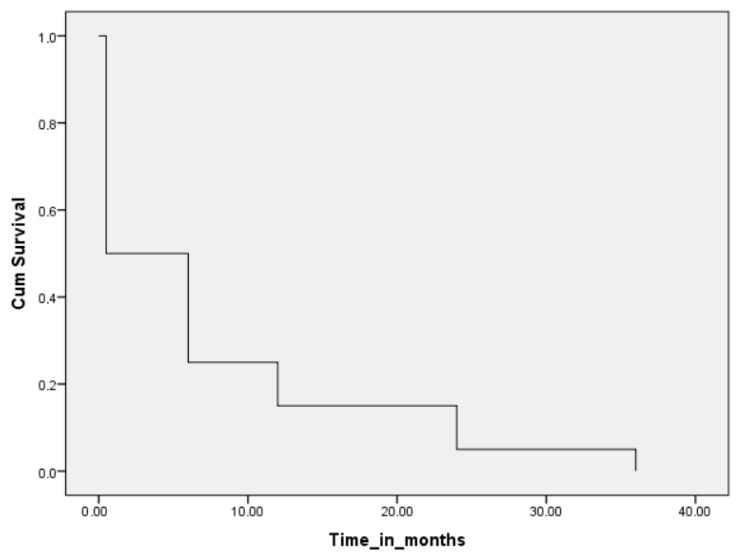
Cox regression analysis for survival of patients.

Furthermore, during the follow-up period of 2021–2024, adverse events (cardiovascular and non-cardiovascular) occurred in a total of 41 patients, and 32 patients were hospitalized, with 16 due to cardiovascular etiology and another 16 due to other etiologies. The primary reason for admission to the cardiology department during the follow-up period was CAD, both acute and chronic coronary syndrome. The most common non-cardiovascular reasons for hospitalization were tumors and chronic obstructive pulmonary disease (Table 6). Another 10 patients (10.1%) died during the follow-up period. Five of them died in 2021, two in 2022, two in 2023, and one in 2024. Figure 1 presents the results of Cox regression analysis for the survival of patients, while the hazard ratios (HRs) and corresponding 95% Confidence Intervals (CIs) are presented in Supplementary Table S1.

## 4. Discussion

Our study included a total of 99 consecutive subjects hospitalized due to moderate and severe forms of COVID-19 pneumonia in “Zvezdara”—University Medical Center in the period 18 March–18 April 2021. The study population consisted mainly of males (male-to-female ratio of 2:1). The potential reason for this distribution between the sexes is interpreted by Jaillon et al. [16], as a consequence of stronger innate and adaptive immune responses in females, so they are generally less sensitive to both bacterial and viral infections.

In our patients, among the symptoms reported during the history taking during hospital admission, malaise was the most common—95%—and was followed by elevated temperature—86%—and dyspnea—47%. In terms of co-morbidity in our cohort, hypertension (present in 84% of cases) and CAD (in 34% of cases) were predominant. Diabetes was present in a quarter of patients (25.5%), as well as the number of active smokers, at 25.3%. In a large meta-analysis by Baradaran et al. [17], they came to the conclusion that cardiovascular diseases are an independent risk factor for a worse outcome in the treatment of patients with COVID-19 pneumonia, and age and male sex had a significant influence on the outcome.

In patients suspected of developing cardiovascular complications (66 patients), an ultrasound of the heart was performed. The majority—42 patients—were in the group with preserved ejection fraction, while 24 had mildly reduced or reduced EF. Twelve patients within the reduced (mildly reduced) group were patients with previous heart disease—CAD with stents and CABG—and three patients had acute myocardial infarction and new changes in myocardial kinetics. For the remaining patients with reduced EF and global hypocontractility, we are not sure whether these changes already existed or if they are new changes that occurred as a result of acute COVID-19 infection. It should be noted that a majority of the assessment of the overall ejection fraction of the heart is subjectively estimated by experienced echocardiographers. The main reason for the examination was clinical orientation and focus on suspicious changes, with the aim of making a decision for the inclusion or correction of existing therapy. If we look at the values of the right ventricle systolic pressure, it was elevated in 23 patients, with most in the mild range. The majority—seven patients with moderate and severe pulmonary hypertension—already had these changes due to recurrent pulmonary embolism, heart failure, and uncorrected valvular disease. If we take into account that our population is predominantly old, and that elevated right ventricle systolic pressure values can be expected, especially in a mild range, we cannot say with certainty that COVID-19 pneumonia is the reason for these findings, as some studies have shown [18,19]. The third finding on cardiac echo was pericardial effusion in six patients as an incidental finding on heart ultrasound, without accompanying typical clinical or ECG changes. Effusions were minor to mild without warning signs of tamponade, so there was no need for pericardial punction.

New cardiovascular events occurred in 47% of patients during hospitalization. The main events were pulmonary thromboembolism at 15.2% and arrhythmia at 12.1%, while CAD, heart failure, and cerebrovascular insult were much less common. Our results were confirmed by other studies [20,21]. Pulmonary embolism was present in 15 patients (6 of them had a prior history of pulmonary embolism), while 12 patients had arrhythmia (8 of them for the first time). It is not surprising if we know that cytokine storms can disrupt coagulation, leading to fibrinolytic imbalance, while simultaneously increasing inflammation contributes to the risk of arrhythmias [22]. Lung CT showed only one proximal occlusion, while the others were distal (segmental or subsegmental) occlusions, and they had a dominantly diffuse type of pneumonia in 96%. Acute myocardial infarction was present in four patients; three underwent coronary angiography with stent implantation. Two patients had a history of coronary disease; this suggests that the incidence of acute coronary disease was the same among coronary patients and the others in our cohort. We could then conclude that the COVID-19 pneumonia was an independent factor for acute myocardial infarction. The exact opposite has been established in a large study by El-Menyar et al. with 16,903 patients, in which patients with previous infarction developed a new infarction in 92.5% of cases [23]. This difference is certainly caused by the huge difference in the number of observed patients. In our cohort, heart failure was most often an exacerbation of previous heart insufficiency. The presence of diabetes can be considered a potential predictor of new heart failure, as it increases the chance of new heart failure almost seven times in our group. There are studies that confirm the positive connection between diabetes and the occurrence or worsening of heart failure [24,25].

The mortality of respondents hospitalized due to COVID-19 pneumonia was 10.1%. This result of ours does not deviate significantly from the results of other studies, where the frequency of lethal outcomes ranges from 5 to 14%, depending on whether the study included moderately severe patients or those in the intensive care unit [21,26,27]. One of the aims of our study was to examine the relationship, that is, the influence of new cardiovascular events on the death outcome. The emerging cardiovascular event with the greatest effect on the prediction of death was new-onset CAD, followed by heart failure and hyperkaliemia. This could be explained by the fact that new cardiovascular complications occur in more severe forms of COVID-19 pneumonia as a reflection of a higher degree of systemic inflammation, tissue stress, and hypoxia, as well as that they occur more often in patients with pre-existing cardiovascular diseases. In isolated hyperkalemia, in addition to the mentioned mechanisms, it may also be a consequence of applied therapy.

All previously treated and discharged patients were evaluated again after three years. Patients’ late symptoms of COVID-19 in the form of extreme malaise and sleepiness, shortness of breath, and palpitations, occurred in 21% of patients, more often in men. The most common symptom was extreme malaise. During the period of April 2021–December 2024, the total mortality was 10.1%. The highest rate of mortality was observed during the first year after discharge from the hospital, at 5%. In the following period, mortality was reduced to 2%. Our results were confirmed by a large study by Ramzi and associates [28], which included a systematic review and meta-analysis of 82 studies. The highest risk of death was observed after one month and was 7.8%, as well as after one year, when it was 7.5%. During 2021–2024, 32.4% of patients were hospitalized again, and 16% of patients were hospitalized due to an acute cardiac condition. The main reason was CAD with 7%, then heart failure with 3%. In our cohort, acute myocardial infarction and heart failure during the follow-up period were more common in patients without a history of these conditions. This leads us to the conclusion that all patients hospitalized for COVID-19 pneumonia have an increased risk of acute coronary events and heart failure after discharge, which has been confirmed by other studies too [29].

Certain limitations of our study should be kept in mind during the interpretation of the results. Our study would have benefited from a larger sample size, i.e., the number of included patients. Some diagnostic procedures, such as echocardiography and lung CT scan, were not performed in all patients, but according to clinical indications. Further, patients with subclinical cardiovascular events were not included, so we do not have an adequate group to compare the obtained results. We should not forget that potential complications of the disease, as well as a fatal outcome, may also be a consequence of the administered therapy—antiviral, corticosteroid, or immunomodulatory. As SARS-CoV-2 is a novel virus (it was initially identified in late 2019), follow-up of infected individuals has only been for a few years at this moment. Also, our follow-up was not continuous; patients were evaluated only once after three years. Studies with a longer follow-up period and more complex analyses of infected patients are needed in order to detect changes and propose adequate treatment in earlier stages of the disease to prevent the multiorgan dysfunction and potential disability of the patients. These studies are also necessary in order to reveal the assumed latent effects.

## 5. Conclusions

Cardiovascular complications are common in patients hospitalized with COVID-19 pneumonia and significantly impact in-hospital outcomes. Post-discharge, these patients remain at increased risk of mortality, particularly within the first year, as well as a high risk of rehospitalization, most often due to acute cardiovascular events such as coronary artery disease and heart failure. These findings underscore the importance of systematic cardiovascular monitoring following initial hospitalization. Furthermore, they highlight the critical role of primary prevention of SARS-CoV-2 infection in reducing the overall burden of long-term complications.

## Figures and Tables

**Table 1:** Distribution of cardiovascular risk factors in participants at hospital admission.

Risk Factor	*n* (%)
Body mass index, mean ± sd	27.7 ± 4.3
Number of smokers	25 (25.3)
Hypertension	83 (83.8)
Heart failure	14 (14.1)
Coronary artery disease	34 (34.3)
Prior revascularization-PCI	19 (19.2)
Prior revascularization-CABG	9(9.2)
Arrhythmia	19 (19.2)
Conduction disorders	8 (8.1)
Pericardial effusion	7 (7.1)
Pulmonary thromboembolism	6 (6.1)
Deep venous thrombosis	2 (2.0)
Type 2 diabetes	25 (25.5)

PCI—Percutaneous Coronary Intervention; CABG—Coronary Artery Bypass Grafting.

**Table 2:** Heart ultrasound parameters of the participants.

Parameter	Mean ± Standard Deviation
Left ventricular systolic dimension (mm)	40.00 ± 7.17
Left ventricular dyastolic dimension (mm)	48.17 ± 8.92
Left atrial dimension (mm)	38.79 ± 4.71
Right ventricle dimension (mm)	27.73 ± 5.54
Right ventricle systolic pressure (mm Hg)	34.77 ± 8.29 (range 22–56)
Ejection fraction (%)	48.53 ± 5.24
Right ventricle systolic pressure	
Normal (≤35 mmHg)	43 patients
Mild (36–40 mmHg)	12 patients
Moderate (41–55 mmHg)	9 patients
Severe (≥56 mmHg)	2 patients
Ejection fraction	
Preserved (≥50%)	42 patients
Mildly reduced (41–49%)	9 patients
Reduced (≤40%)	15 patients

**Table 3:** Frequency of new cardiovascular events among hospitalized COVID-19 pneumonia patients.

New Acute Cardiovascular Event	*n* (%)
Heart failure	6 (6.1)
Acute myocardial infarction	4 (4.0)
Pericardial effusion	6 (6.1)
Arrhythmia:	12 (12.1)
- Atrial fibrillation	8 (8.0)
- Extrasystolic arrhythmia	4 (4.0)
Pulmonary thromboembolism	15 (15.2)
Deep venous thrombosis	1 (1.0)
Cerebrovascular insult	3 (3.0)

**Table 4:** Significant predictors of intrahospital mortality.

Parameter	B	OR	95% CI	*p*-Value
Increased leukocyte count	1.743	5.714	1.37–23.81	0.017
Platelets	−0.016	0.984	0.97–0.99	0.007
Decreased platelets	2.037	7.667	2.07–28.34	0.002
C-reactive protein	0.012	1.012	1.0–1.02	0.014
Ferritin	0.001	1.001	1.00–1.01	0.002
Total cholesterol	−2.643	0.071	0.02–0.28	<0.001
Triglycerides	−2.309	0.099	0.02–0.43	0.002
LDL	−2.007	0.134	0.03–0.69	0.017
Troponin I	2.234	9.333	1.63–53.27	0.012
Glucose level	0.680	1.974	1.35–2.89	<0.001
Increased glucose level	2.037	7.667	2.07–28.34	0.002
HbA1c	1.933	6.909	1.89–25.26	0.003
Urea	0.177	1.194	1.08–1.32	<0.001
Creatinine	0.012	1.013	1.01–1.02	0.002
Increased creatinine	1.933	6.909	1.89–25.26	0.003
LDH	0.004	1.004	1.01–1.01	0.003
Albumins	−0.155	0.856	0.76–0.96	0.011
Potassium	1.810	6.110	1.75–21.33	0.005
Increased potassium	2.639	14.000	2.65–73.88	0.002
Heart rate	0.076	1.079	1.03–1.12	<0.001
Arrhythmia	1.315	3.724	1.03–13.42	0.044
Heart failure	3.056	21.250	3.36–134.56	0.001
Coronary heart disease	3.356	28.667	2.69–305.14	0.005
Pulmonary embolism	2.234	9.333	1.63–53.27	0.012
Arrhythmia de novo	2.100	8.163	2.05–32.56	0.003

LDL—Low-density lipoprotein; HbA1c—hemoglobin A1C; LDH—Lactate dehydrogenase.

**Table 5:** Frequency of late COVID symptoms.

Symptom	*n* (%)
Short breath	11 (11.1)
Palpitations	9 (9.1)
Extreme malaise and sleepiness	21 (21.2)

**Table 6:** Adverse events during the follow-up period.

Adverse Events During the Follow-Up Period	*n*
Acute myocardial infarction	2
Chronic coronary syndrome	5
Heart failure	4
Atrial fibrillation	2
Atrioventricular block gradus III	1
Pulmonary embolism	2
Cerebrovascular insult	2
COPD	11
Type 2 diabetes	4
Cancer	8

COPD—chronic obstructive pulmonary disease.

## Data Availability

The data presented in this study are available on request from the corresponding author due to privacy concerns.

## Supplementary Materials

The following supporting information can be downloaded at: https://www.mdpi.com/article/10.3390/v17101293/s1, Table S1: Results of Cox proportionate regression model for mortality during study period.

## Abbreviations

The following abbreviations are used in this manuscript:

ACE2	Angiotensin-converting enzyme 2
PCR	Polymerase chain reaction
CT	Computed tomography
ECG	Electrocardiogram
BNP-s	Brain natriuretic peptides
PCI	Percutaneous Coronary Intervention
CABG	Coronary Artery Bypass Grafting
LDL	Low-density lipoprotein
HbA1c	Hemoglobin A1C
LDH	Lactate dehydrogenase
COPD	Chronic obstructive pulmonary disease
RVD	Right ventricular dysfunction
