# Supplementary material for: COVID-19 and Cardiovascular Complications: A Follow-Up Study from Tertiary Center
Source: Viruses. 2025 Sep 24;17(10):1293. doi: 10.3390/v17101293 (PMC12568308; doi:10.3390/v17101293)
Supplement: Supplementary file 1 [file viruses-17-01293-s001.zip › viruses-3849559-supplementary.pdf]

Supplementary Table S1. Results of Cox proportionate regression model for mortality during study period

Variable	Hazard ratio	95% Confidence interval	p-value
Age	1.001	0.95-1.06	0.967
Sex	1.246	0.49-3.18	0.645
Mechanical ventilation	1.325	0.47-3.76	0.597
Severity of pneumonia	1.049	0.60-1.84	0.866
Elevated d-dimer	0.891	0.36-2.22	0.804
Right ventricle systolic pressure	1.024	0.95-1.11	0.549
Ejection fraction	0.993	0.96-1.03	0.682
Previous coronary heart disease	1.251	0.35-4.45	0.729
Previous heart failure	1.480	0.56-3.91	0.429
